# The Treatment of Congenital Equinovarus during Early Infancy
1Lancet, July 9, 1904.


**Published:** 1904-07-23

**Authors:** 


### THE TREATMENT OF CONGENITAL EQUINOVARUS DURING EARLY INFANCY.[1]

Congenital talipes equinovarus is treated in many different ways with a varying degree of success largely dependent upon the age at which treatment is commenced and the conscientiousness with which it is carried out. In the case of infants suffering from this deformity the best method, according to Laming Evans,[1] is one which he has found successful in all but a very small minority of his cases, and which has the merit of being easy of application and rapid. He corrects the varus first and thus converts a compound into a simple deformity, later he deals with the equinus, and last of all the inward rotation of the limb.

The tibialis anticus and posticus with the flexor longus digitorum of the affected limb are divided above the level of the ankle-joint. This operation should be performed at the earliest possible age, and chloroform is unnecessary. The varus curve is immediately rectified and the foot is bandaged to a malleable iron splint in an improved position. A course of manipulation is commenced as soon as the puncture has healed gradually varying the shape of the malleable splint until at the end of about thirty days the varus is corrected or over-corrected at the mid-tarsal joint.

The second stage begins with division of the tendo Achillis, immediate rectification of the equinus and subsequent manipulations. During the intervals the foot is retained in position in a padded tin shoe consisting of a footpiece fixed to a legpiece so that the foot is held in a position of dorsiflexion and slight eversion.

It is well at this stage to commence by manipulation to correct the inward rotation of the leg which takes place at the hip joint. When the deformity of the foot has been corrected, daily manipulation, with constant use of some sort of walking apparatus, is a necessity. Cure is not attained till adult life is reached.

The main advantages of a malleable iron splint over plaster-of-Paris are:—(1) manipulations can be carried on at home in the intervals of attending the surgeon; (2) a soiled splint can easily be replaced; (3) the mechanical advantage is greater; and (4) the risk of excoriation of the skin is diminished.

The arguments against early treatment and in favour of a major operation on the tarsal bones when childhood is well advanced, are the dangers of chloroform in infancy, the risk of ulceration by plaster-of-Paris, or softening of the latter by urine, and the long course of treatment.

In Evans's series of 14 cases neither chloroform nor plaster-of-Paris were needed.

[1] Lancet, July 9, 1904.

